# Transcriptome meta-analysis reveals a dysregulation in extra cellular matrix and cell junction associated gene signatures during Dengue virus infection

**DOI:** 10.1038/srep33752

**Published:** 2016-09-21

**Authors:** Sumbul Afroz, Jeevan Giddaluru, Mohd. Manzar Abbas, Nooruddin Khan

**Affiliations:** 1 School of Life Sciences, Department of Biotechnology and Bioinformatics, University of Hyderabad, Hyderabad-500046, Telangana, India

## Abstract

Dengue Viruses (DENVs) cause one of the most prevalent arthropod-borne viral diseases affecting millions of people worldwide. Identification of genes involved in DENV pathogenesis would help in deciphering molecular mechanisms responsible for the disease progression. Here, we carried out a meta-analysis of publicly available gene expression data of dengue patients and further validated the meta-profile using *in-vitro* infection in THP-1 cells. Our findings reveal that DENV infection modulates expression of several genes and signalling pathways including interferons, detoxification of ROS and viral assembly. Interestingly, we have identified novel gene signatures comprising of INADL/PATJ and CRTAP (Cartilage Associated Protein), which were significantly down-regulated across all patient data sets as well as in DENV infected THP-1 cells. PATJ and CRTAP genes are involved in maintaining cell junction integrity and collagen assembly (extracellular matrix component) respectively, which together play a crucial role in cell-cell adhesion. Our results categorically reveal that overexpression of CRTAP and PATJ genes restrict DENV infection, thereby suggesting a critical role of these genes in DENV pathogenesis. Conclusively, these findings emphasize the utility of meta-analysis approach in identifying novel gene signatures that might provide mechanistic insights into disease pathogenesis and possibly lead towards the development of better therapeutic interventions.

Dengue virus infection is emerging expeditiously as a global health problem with the arthropod-borne flavivirus infecting 50–100 million people annually^1^. Dengue infection in majority cases may be asymptomatic or may result in a wide case of clinical symptoms^2^, which range from mild flu- like syndrome developing self-limiting febrile illness known as Dengue fever (DF), to the more aggravated and severe form of disease characterized by coagulopathy, increased vascular fragility and thrombocytopenia accompanied with excessive plasma leakage known as Dengue Haemorrhagic Fever (DHF)^3^. DHF may progress to hypovolemic shock called Dengue Shock Syndrome (DSS)^3^,^4^. DHF/DSS occur mostly in individuals reported with secondary DENV infection^5^, and has been attributed to the phenomenon of antibody-dependent enhancement (ADE) of viruses^6^. According to WHO, the constant upsurge in DENV cases may be ascribed to the geographical expansion of both vector and virus with the emergence of hyperendemicity in many urban areas of tropics, absence of proper anti-viral therapy and licensed DENV vaccine^1^. Moreover, the success of DENV could be partly attributed to the poor understanding of complex host-virus interaction, concerns about the role of the host protective immune responses contributing to the disease outcome and severity^6^, and unavailability of an appropriate animal model^7^. Although unprecedented efforts have been made to identify multitude of factors which have foremost insinuations in DENV pathogenesis, including viral load^8^, host genetic predisposition^7^, autoantibodies generation^6^, and T-cell activation leading to enhanced cytokine expression including Tumor necrosis factors (TNF), interleukin(IL)-1, IL-2 and IL-6, production of chemical mediators, platelet-activating factor (PAF), complement components C3a and C5a, and histamines^9^, yet there is a dearth of knowledge about various genes, and genomic networks associated with the disease. Recent advances in microarray technology have fuelled high-throughput whole transcriptome analysis of DENV infected individuals, thus helping in understanding the biology of DENV infection. Using such technology, several pathways including NF-ĸβ^10^, type-I interferon^3^, ubiquitin proteasome and ER stress pathway^10^ have been associated with DENV pathogenesis. Despite the implementation of numerous dengue microarray experiment, there exists variability in gene expression profiles across various studies. A rational approach to expunge this variability in transcriptome profiles is to effectively analyse, compare and integrate multiple microarray experiment data using meta-analysis approach, which assists researchers to overcome limitations of single microarray experiments having small sample size, compromised statistical power and inconsistent gene expression data^11^. Also, the meta-analysis of microarray data has been an effective strategy to compare results across different microarray platforms, thus resulting in the generation of a robust and reliable gene expression data^12^. Such approaches were initially implemented using prostate cancer patient data, which revealed a significant dysregulation of polyamine and purine biosynthesis pathways^13^. Lately, many microarray meta-analysis methods have been introduced to extract novel gene expression signatures from multiple microarray data^14^. Although such approaches have been extensively used in oncology^15^ and infection^16^, they have not yet, to our knowledge applied to DENV infection.

Here, we have implemented a meta-analysis method on publicly available dengue patient microarray data sets. At first, the data sets were prepared and analysed separately to check for differential expression of genes (DEGs) between the diseased (DF, DHF/DSS) and healthy groups. DEGs obtained from these analysed data sets were overlapped, and common DEGs were extracted, resulting in a robust, consensus meta-gene expression profile of DENV patients. Meta-analysis findings were validated by examining the expression levels of some selected genes in DENV infected THP-1 cells through RT-qPCR. Later, we have tried to understand the functionality of the identified genes during DENV infection using gene-overexpression approach. The findings of this study provide novel gene signatures and possible mechanistic insights in the pathogenesis of the disease that might lead towards the development of novel diagnostic and therapeutic interventions against DENV infection.

## Results

### Primary Analysis

**Figure 1:**
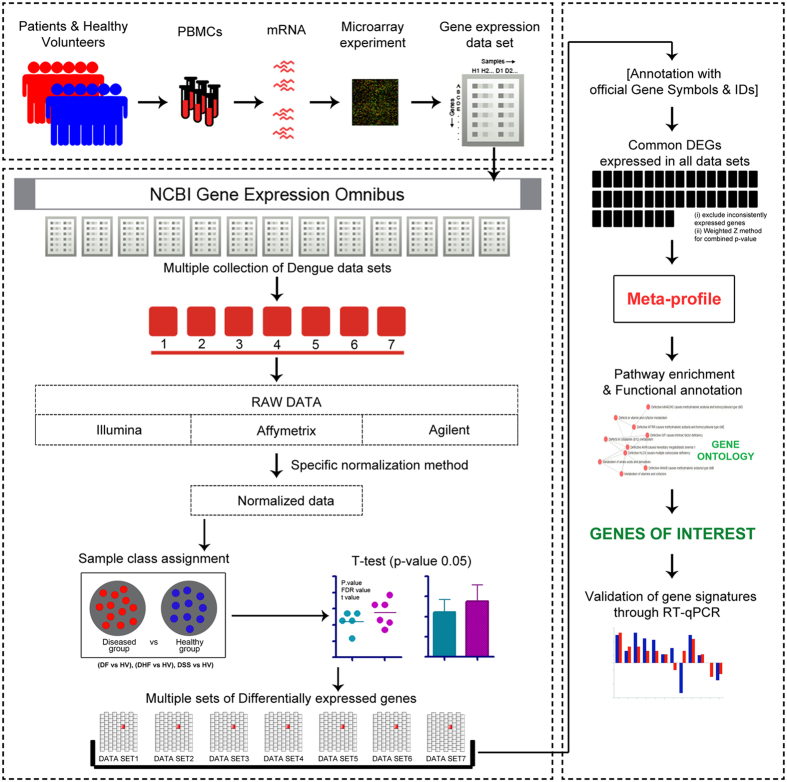
Pictorial representation of the meta-analysis approach adopted for this study.

In our study, four dengue microarray data sets (GSE43777, GSE51808, GSE17924 and GSE16463) which fulfilled our inclusion and exclusion criteria (described in Materials and Methods) were taken. Depending on disease category (DF or DHF/DSS), data sets were further manually curated into seven custom data sets (Supplementary Table S1 provides the details of custom data sets). Analysis of each data set was performed for diseased (DF or DHF/DSS) versus respective healthy volunteer (HV) group to check for differential expression in each gene using a student’s t-test with a particular threshold (p-value < 0.05). Later, p-values of the genes in each data set were adjusted using Benjamini and Hochberg’s method to control the false discovery rate (FDR)^17^. In total 3,57,922 gene expression measurements were computed for 132 dengue infected patients and 71 healthy volunteers, out of which 87,523 gene measurements showed up a significant change in expression. The primary analyses of these individual data sets generated 7 sets of differentially expressed genes (DEGs) which were further assigned for our meta-analysis study. A detailed flow chart of our work study is shown in Fig. 1.

### Meta-Analysis

**Figure 2:**
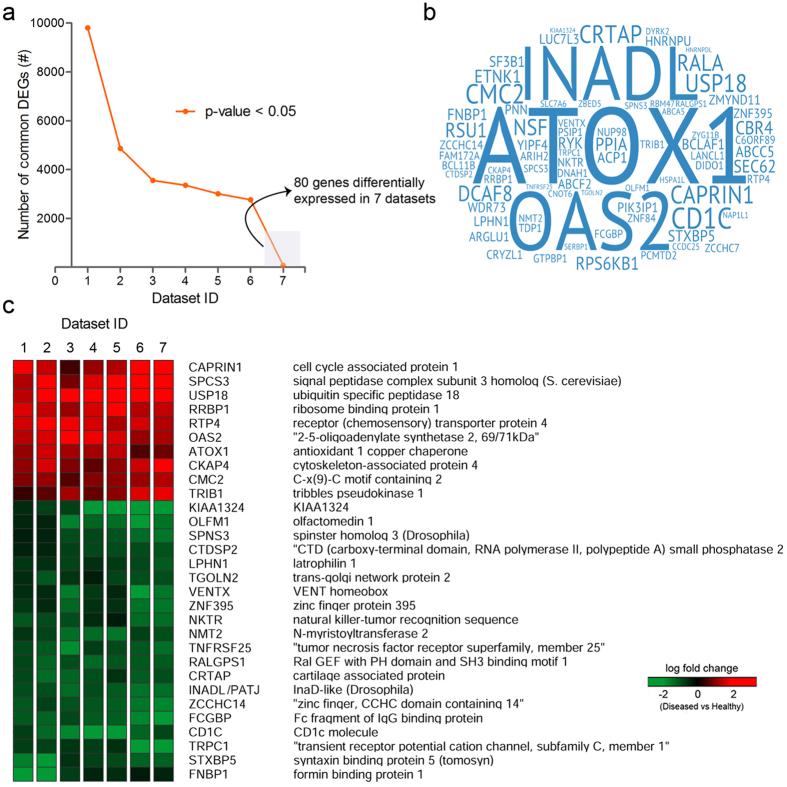
Transcriptome meta-analysis of Dengue virus infected patients. (**a**) A graph representing data sets sharing the number of common DEGs. (**b**) Cloud representation of common DEGs expressed across 7 data sets. (**c**) Heat map showing consistently expressed genes (meta-genes) in different data sets used in this study.

Our selected data sets majorly corresponded to three different platforms; Affymetrix, Agilent, and Illumina. Depending on the type of platform, raw expression data of each data set was pre-processed using an optimal normalization method, enabling a maximum background correction. Subsequently, a meta-analysis was performed to extract intersected DEGs (common DEGs) across multiple sets of DEGs obtained during our primary analyses. 80 DEGs were found to be commonly expressed in all data sets (Fig. 2a,b). Out of the 80 common DEGs, only 30 were consistently expressed (either completely up-regulated or down-regulated) across all data sets (Fig. 2c). Independent q-values (adjusted p-values) of these consistently expressed DEGs from each data set were computed for combined p-values using the weighted Z-method^18^ and were found to be satisfied as per the assigned threshold (<0.05). These consistently expressed genes (10 up-regulated and 20 down-regulated) were finally selected as our meta-profile (Fig. 2c) and ranked according to the log fold change score (Supplementary Table S2). The up-regulated meta-genes include CAPRIN1, SPCS3, USP18, RRBP1, RTP4, OAS2, ATOX1, CKAP4, CMC2, TRIB1, whereas KIAA1324, OLFM1, SPNS3, CTDSP2, LPHN1, TGOLN2, VENTX, ZNF395, NKTR, NMT2, TNFRSF25, RALGPS1, CRTAP, INADL/PATJ, ZCCHC14, FCGBP, CD1C, TRPC1, STXBP5, FNBP1 were found to be down-regulated (Fig. 2c).

### Functional Annotation

To interpret the functional roles of the meta-genes, a GO enrichment analysis was performed using Enrichr^19^, a web-based tool based on Gene Ontology. Based on the input set of genes, top enriched GO Biological processes included peptidyl-proline modification (GO: 0018208), type 1 interferon signalling pathway (GO: 0060337), cellular response to type 1 interferon (GO: 0071357), protein lipidation (GO: 0006497), macro autophagy (GO: 0016236), regulation of beta-amyloid formation (GO: 1902003), cellular copper ion homeostasis (GO: 0006878) (see Supplementary Fig. S1). GO Molecular functions; peptide binding (GO: 0042277), amide binding (GO: 0033218), ligase regulator activity (GO: 0055103), inositol 145 triphosphate binding (GO: 0070679), tumor necrosis factor-activated receptor activity (GO: 0005031) were found to be enriched in the analysis (see Supplementary Fig. S2).

### Pathway Enrichment Analysis

To explore the involvement of candidate meta-genes in pathways, a pathway enrichment analysis was performed using Reactome FI plug-in (Cytoscape)^20^, based on Reactome pathway database. 10 out of the 30 candidate signatures were observed to be directly linked to the pathways of Reactome pathway library. OAS2 and USP18 were found to be significantly enriched in Interferon alpha/beta signalling pathway and NMT2 in Synthesis & processing of GAG, GAGPOL polyproteins, and assembly of the HIV virion. Hit genes also include ATOX1 and CRTAP, which were found to be enriched in detoxification of reactive oxygen species and collagen biosynthesis pathways respectively. Extracellular matrix associated pathways such as tight junction interaction and cell-cell junction organizations were enriched for PATJ (see Supplementary Fig. S3 and Supplementary Table S4).

### Validation of the Meta-analysis data

**Figure 3:**
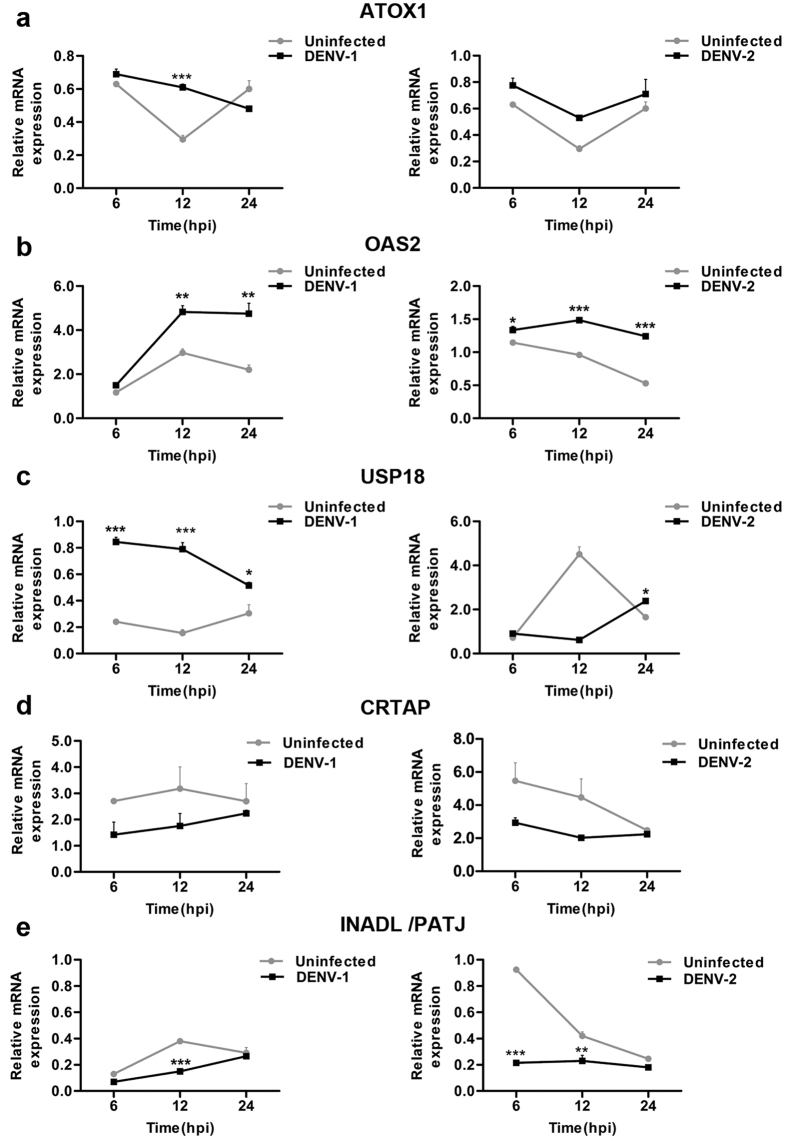
Relative mRNA expression levels of five candidate genes (ATOX1, OAS2, USP18, CRTAP and PATJ) in THP-1 cells infected with DENV. Quantitative Real-time polymerase chain reaction (RT-qPCR) was carried out to quantify the relative expression of the above-mentioned candidate genes in THP-1 cells infected with DENV-1 (Hawaii) and DENV-2 (TR1751) at a multiplicity of infection (moi) of 5 for the indicated time points. The relative expression of each gene (**a**) ATOX1, (**b**) OAS2, (**c**) USP18 (**d**) CRTAP, (**e**) PATJ was normalized to housekeeping gene β-Actin. Results are representative of one of three independent experiments and the error bars represent the standard error of the means (SEM). P values were determined based on comparison with uninfected cells. Statistical analysis was performed using two-way ANOVA with Bonferroni post-hoc test to identify significance using Graph Pad Prism5 software. ***P < 0.001, **P < 0.01, *P < 0.05 were considered statistically significant.

**Figure 4:**
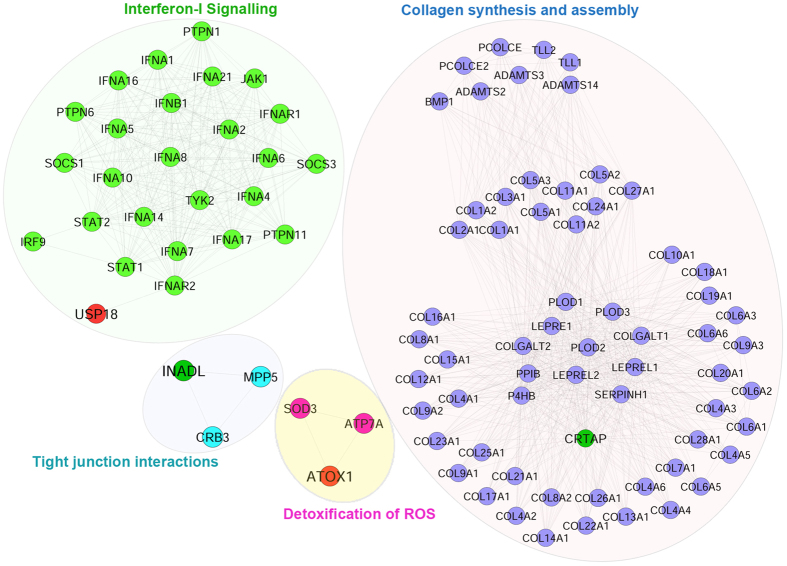
Pathway networks of candidate genes that were validated in Dengue infected patient samples. Pathway networks of genes USP18, CRTAP, ATOX1, PATJ were generated based on Reactome Pathway database using Cytoscape software. No pathway interactions were found for OAS2. Target genes with red colour indicate upregulation; while with green colour indicate down-regulation.

Pathway enrichment analysis of the meta-gene profile revealed a total of ten genes which were found to be associated with distinct pathways. Expression levels of five pathway associated genes, three upregulated (ATOX1, OAS2, and USP18) and two down-regulated (CRTAP, PATJ) genes in the meta-analysis result were selected for validation in DENV infected THP-1 monocytic cell line through quantitative real-time-PCR (RT-qPCR). Relative expressions of these meta-genes were monitored in THP-1 cells infected with DENV-1 (moi 5) and DENV-2 (moi 5) for different time points (6–24 hours). Interestingly, our RT-qPCR data reveals that the meta-genes ATOX1, OAS2 and USP18 were consistently up-regulated with a distinct pattern with infection time-course in THP-1 cells infected with DENV-1 or DENV-2 (Fig. 3a–c). Concomitant to our meta-analysis results, we observed a relative dearth of extracellular matrix related (CRTAP) and tight-junction associated (PATJ) mRNA levels in both DENV-1 and DENV-2 infected THP-1 cells compared to uninfected mock controls (Fig. 3d,e). Despite few differences in the expression level of the candidate genes at some time points, overall these results correspond well with our meta-analysis findings, stipulating the credibility of our approach to identify differentially expressed genes during DENV infection. Further, pathway networks of these validated candidate signatures were generated and visualized using Cytoscape software (Fig. 4).

### Functional characterization of ECM-associated meta-gene signatures comprising of PATJ and CRTAP reveals a crucial role of these genes in restricting DENV infection

**Figure 5:**
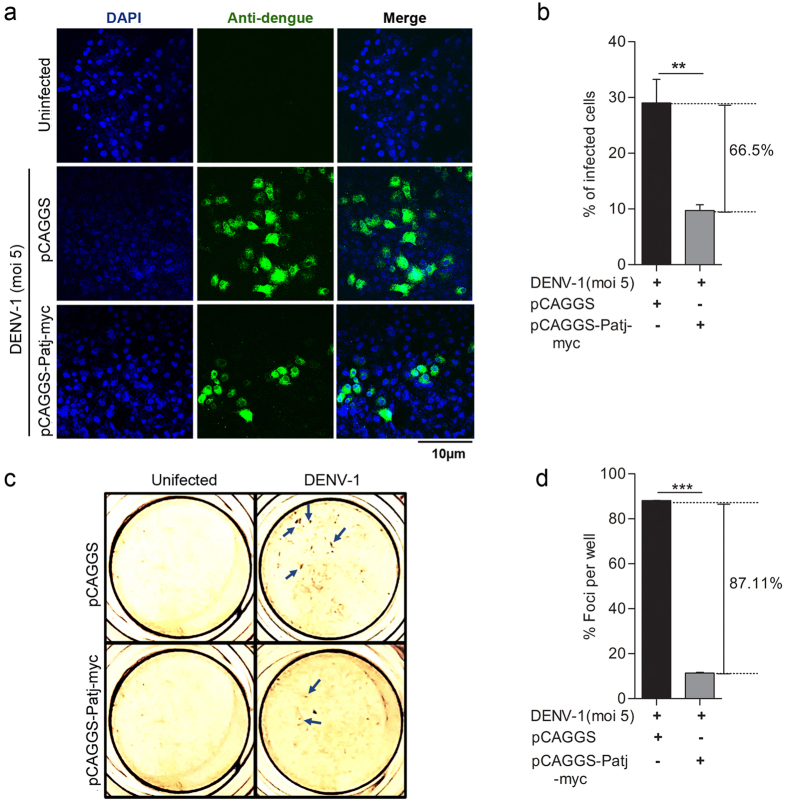
PATJ overexpression interferes with DENV-1 infection. (**a**) Laser scanning confocal microscopy to visualize the number of DENV-1 infected Vero cells transfected with either pCAGGS (vector control) or pCAGGS-Patj-myc. Vero cells were transfected with equal concentrations of both the plasmids for 36 hours followed by infection of DENV-1 (moi 5) for another 18 hrs. Following incubation, the cells were fixed, permeabilized, and stained with anti-dengue monoclonal Ab (green). (**b**) Quantification of the number of DENV-1 infected cells in vector transfected and pCAGGS-Patj-myc transfected Vero cells. Infected cells were counted in at least ten different fields for each experimental condition using Image J software and the average number of infected cells per field were plotted. (**c**) Foci forming unit reduction assay (FFURA) on vector transfected and pCAGGS-Patj-myc transfected Vero cells was performed to determine the antiviral activity of PATJ on DENV-1 using DENV foci immunostaining method as described in Materials and Methods on the fourth day after infection. Arrows indicate representative DENV-1 foci. (**d**) Data from duplicate assays of three independent experiments were plotted. The percentage of foci reduction is represented in the graph. Statistical analysis was performed using Student’s t-test using Graph Pad Prism version 5 (Graph Pad Software Inc., San Diego, CA.). Error bars represent the standard error of the mean (SEM). ***P < 0.001, **P < 0.01, *P < 0.05 were considered statistically significant.

**Figure 6:**
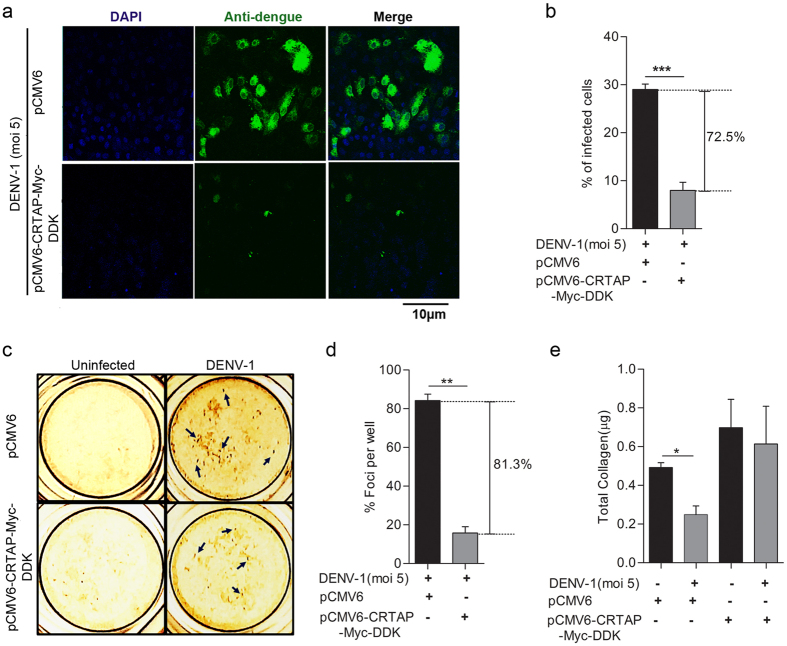
Over-expression of CRTAP gene inversely regulates DENV-1 infection. (**a**) Laser scanning confocal microscopy to visualize the number of DENV-1 infected Vero cells transfected with either pCMV6 (vector control) or pCMV6-CRTAP-Myc-DDK. Vero cells were transfected with equal concentrations of both the plasmids for 36 hours followed by infection of DENV-1 (moi 5) for another 18 hrs. Following incubation, the cells were fixed, permeabilized, and stained with anti-Dengue monoclonal Ab (green). (**b**) Quantification of the number of DENV-1 infected cells in vector transfected and pCMV6-CRTAP-Myc-DDK transfected Vero cells. Infected cells were counted as mentioned in the previous figure. (**c**) Foci forming unit reduction assay (FFURA) on vector transfected and pCMV6-CRTAP-Myc-DDK transfected Vero cells was performed to determine the antiviral activity of CRTAP on DENV-1 as already described in material and methods. Arrows indicate representative DENV-1 foci. (**d**) Data from duplicate assays of three independent experiments were plotted. The percentage of foci reduction is represented in the graph. (**e**) Collagen estimation was done in vector transfected or pCMV6-CRTAP-Myc-DDK transfected cell lysates as described in material and methods. DENV-1 infection was given as indicated. Statistical analysis was performed using Student’s t-test using Graph Pad Prism version 5 (Graph Pad Software Inc., San Diego, CA.). Error bars represent the standard error of the mean (SEM). ***P < 0.001, **P < 0.01, *P < 0.05 were considered statistically significant.

The most prodigious finding that emerged from our meta-analysis study is the identification of novel gene signatures comprising of extracellular matrix (ECM) associated, PATJ (a tight junction protein) and CRTAP (involved in collagen synthesis) genes, which were found to be down-regulated during DENV infection, thereby suggesting the involvement of these genes in perturbing DENV pathogenesis possibly by restricting DENV invasion and transmission. Therefore, we examined the functionality of these gene signatures on DENV pathogenesis by overexpressing these genes in Vero cells followed by DENV infection. Vero cells were transiently transfected with either vector alone or PATJ expressing (pCAGGS-Patj-myc) plasmid for 36 hrs followed by DENV-1 or DENV-2 (moi 5) infection for further 18 hrs. The overall percentage of DENV infected cells were visualized through confocal microscopy. We observed involvement of PATJ in restricting DENV infection, as our results revealed a significant inhibition in DENV-1 (Fig. 5a,b) or DENV-2 (see Supplementary Fig. S4a,b) infection in cells overexpressing PATJ protein as compared to vector controls. The *in vitro* anti-DENV activity of PATJ protein was also evaluated by DENV foci reduction assay, as the cells transfected with pCAGGS-Patj-myc plasmid showed a significant reduction in DENV-1 foci formation as compared to vector transfected Vero cells (Fig. 5c,d). In addition to PATJ, another ECM associated gene that popped-up during our meta-analysis study and found to be down-regulated during DENV infection was CRTAP. Previous studies have reported the involvement of CRTAP in perturbing viral replication^21^, as well as in promoting collagen synthesis^22^. Therefore, next, we examined the level of total collagen during DENV infection in pCMV6-CRTAP-Myc-DDK or vector transfected Vero cells and correlated it directly to DENV pathogenesis in the transfected cells. Akin to PATJ, CRTAP overexpression was also found to inhibit DENV-1 (Fig. 6a,b) or DENV-2 (see Supplementary Fig. S5a,b) infection compared to vector transfected controls as indicated by the percentage of total infected cells. Similar results were observed through DENV foci reduction assay with pCMV6-CRTAP-Myc-DDK plasmid transfected cells showing a significant reduction in DENV-1 foci formation as compared to vector transfected Vero cells (Fig. 6c,d). The level of total collagen was found to be inversely correlated with DENV pathogenesis as our results showed a significant reduction in total collagen levels in DENV infected vector transfected cells compared to uninfected mock controls (Fig. 6e). Conversely, the level of collagen in pCMV6-CRTAP-Myc-DDK transfected cells were comparable in both DENV infected and uninfected controls (Fig. 6e), insinuating that CRTAP overexpression perturbs DENV infection in Vero cells. These findings categorically suggest that DENV suppresses CRTAP and PATJ genes expression, which in turn perturbs collagen synthesis and ECM, thereby influencing DENV pathogenesis.

## Discussion

Microarray technology has been one of the greatest triumphs in modern medicine. This technology has emerged as a robust tool for researchers to identify gene signatures for the development of therapeutic biomarkers for several diseases^23^. Though there exists a considerable difference in performance across various platforms, the primary objective to conduct a microarray experiment is to test for differential expression of genes between two or more groups. Often, the differential expression is checked using well-grounded statistics; T-test for two groups, F-test for more than two groups, assigning a specific threshold (P < 0.05). The statistic test is usually conducted with a null-hypothesis considering there is no differential expression of genes between the groups. Genes with p-values less than assigned threshold (<0.05) reject the null hypothesis (accept alternative hypothesis) indicating differential expression of genes between the assigned groups. Due to multiple testing and the noisy nature of the microarray data, many false positives arise and often are controlled using the Bonferroni correction method^17^. Additionally, deletion of truly differentially expressed genes (false negatives) while adjusting the p-values also contribute to the loss of valuable data during the analysis. Though false negatives can be adjusted variably but not entirely^24^, these variations in gene expression profiles generated after multiple testing can be eliminated by combining multiple microarray data sets. Despite the availability of several DENV microarray data, a consistent transcriptome profile of DENV infected subjects remains elusive. Therefore, we have undertaken a meta-analysis approach to generate a meta-profile across multiple DENV microarray data sets. In our study, the meta-analysis was performed on publicly available DENV microarray data sets, resulting in the identification of a consistent gene expression profile (meta-profile). Each data set was analysed separately to obtain multiple sets of DEGs, which were overlapped, resulting in a consistent gene expression profile with a greater statistical power. Further, this meta-profile was validated *in-vitro* in THP-1 cells infected with DENV through quantitative real-time PCR. The findings of this study categorically manifest the involvement of novel cell-cell interactions and communication pathways, which were not described previously to play a crucial role in DENV pathogenesis. In consent with previous studies which have shown alteration in various aspects of IFN-signalling pathway during DENV infection^25^, our study also points towards perturbations in type–I interferon (type-I IFN) response as indicated by the relative abundance of OAS2 and USP18 gene transcripts during early dengue infection (Figs 2 and 3b,c). OAS2 is one of the isoforms of type-I IFN induced protein OAS (Oligoadenylate synthetases)^26^, which gets activated by viral single or double stranded RNA to catalyze the oligomerization of ATP into 2′,5′-linked oligoadenylate, which further activates latent RNase L^26^. The activated RNase L, in turn, degrades the viral as well as cellular RNAs thereby blocking viral replication^27^. These degraded RNAs further activate various cytosolic Pathogen Recognition Receptors like RIG-1 and MDA-5 resulting in the modulation of IFN signalling^28^. Previous studies have shown upregulation of USP18 gene upon viral infection^29^. USP18 plays a decisive role in impeding type I-Interferon signalling via inhibition of STAT signalling pathway upon viral infection^30^. Moreover, Mouse embryonic fibroblasts (MEFs) and bone marrow derived macrophages isolated from USP18^−/−^ mice show constrained lymphocytic choriomeningitis virus (LCMV) replication^29^, thus highlighting the active role of USP18 gene in promoting viral replication. Further, our study depicts involvement of some other previously unnoticed genes and pathways such as NMT2 (protein lipidation), CTDSP2 (phosphoric- ester hydrolase activity), SPCS3 (peptidase activity), TRPC1 (trans-membrane transportation of divalent inorganic ions) and TGOLN2 (Clathrin derived vesicle budding) during DENV infection.

Various studies have illustrated a vital synergy between the generation of cellular oxidative stress and DENV pathogenesis^31^. DENV infection results in the intracellular accumulation of NADPH-oxidase-dependent Reactive oxygen species (ROS). Increased ROS production promotes NF-Kβ dependent inflammatory responses, IRF3 mediated antiviral responses and, p53 mediated mitochondrial apoptosis of infected cells^31^. Inhibition of ROS diminishes the host innate immune responses thereby facilitating DENV replication. To evade ROS- induced host antiviral responses, DENV stimulates the expression of transcription factors Nrf2, EPAS1, and HIF1 dependent antioxidant genes, which act as ROS scavengers^31^. Our meta-analysis data have identified a previously unnoticed oxidative stress responsive gene ATOX1 (Antioxidant protein 1) during DENV infection, which is significantly upregulated across all the dengue patients PBMC data sets. Further, our RT-qPCR results also reveal an upsurge of ATOX1 gene in DENV infected THP-1 cells at early time points (Figs 2 and 3a). ATOX1 is a metal chaperon which is majorly involved in copper homeostasis^32^. Mutations in this gene have been associated with cancer carcinogenesis^33^,^34^ and 5q syndrome^35^. Its implication in DENV infection is yet to be unearthed.

The accomplishment of viral infection largely depends on the ability of viruses to modulate cell-cell contact and extracellular matrix components (ECM)^36^. The ECM is an incredibly dynamic structure responsible for regulating a wide range of functions including cell proliferation, migration, and differentiation^37^. Dysregulation of ECM composition and abundance, contributes to diverse pathological conditions including fibrosis and invasive cancer^37^. Furthermore, it has been shown that some components of ECM, such as galectin and collagens are involved in the surface transmission of *human T-lymphotropic virus* (HTLV)^38^. Interestingly, our study for the first time have identified gene signatures encompassing, PATJ and CRTAP (Cartilage Associated Protein) genes, which are well-known orchestrator of cell-cell contact and collagen assembly^39^, were found to be respectively down-regulated consistently in all DENV microarray data sets included in our meta-analysis study as well as during *in-vitro* infection of THP-1 cells with DENV (Figs 2 and 3d,e). PATJ is a scaffolding protein which belongs to the family of proteins with multiple PDZ domains^40^, and is found to interact with a subset of other cell junction proteins like claudins^41^,^42^. PATJ is majorly involved in maintaining the integrity and polarity of tight junctions and may also regulate protein targeting to various organelles. Recent studies have documented multiple PDZ containing tight junction membrane proteins, to play a major role in viral adherence and entry into the host cells^43^. West Nile virus (WNV) infects epithelial and endothelial cells through GTPase-dependent endocytosis of Claudin -1 and JAM-1 proteins, thereby accelerating the degradation of lysosomal proteins in the polarized cells^43^. High-risk human papillomavirus E6 protein also interacts and degrades an assorted number of PDZ proteins most importantly PATJ, thereby effecting assembly of tight junctions in polarized epithelial cells^44^. Additionally, the HMEC-1 endothelial cell line was found to be infected by DENV serotype-2 through the displacement of related occludin protein from the tight junction complex^45^. Apart from PATJ, the other gene that popped-up during meta-analysis was CRTAP gene which has been previously stated to play an important role in type II and type VII forms of recessive Osteogenesis Imperfecta, a rare form of the disease^46^. CRTAP protein associates with two other proteins namely leprecan, a basement membrane-associated proteoglycan^47^ which has collagen prolyl 3-hydroxylase (P3H) activity^48^ and cyclophilin B^39^, in Endoplasmic Reticulum forming a trimeric complex. This trimeric complex is believed to be a requisite for the proline 3-hydroxylation which is one of the major post-translational modification event, critical for the appropriate folding and assembly of collagen, found abundantly in extracellular matrix^46^. The abundance of CRTAP transcripts has been reported to be negatively associated with HIV virus replication^21^. Our finding categorically reveals that CRTAP gene expression is down-regulated in DENV patients. The functional elucidation of PATJ and CRTAP genes categorically demonstrate that they have a vital role in restricting DENV infection (Figs 5 and 6), however, correlation of this phenomenon with the disease severity and outcome needs to be completely deciphered. Having observed the functionality of these genes, it is tempting to speculate that down-regulation of the gene signatures comprising of PATJ and CRTAP might have an implication in DENV pathogenesis, possibly by altering virus penetration and permeability of target cells, which has been propounded to be a major factor in plasma leakage, a hallmark of Dengue Haemorrhagic fever. Nevertheless, several points need to be addressed in the future to investigate the role of these genes/pathways in modulating the molecular invasion of DENV imperative to predict the clinical outcome of the disease in infected patients which could usher towards the development of novel therapeutic interventions against DENV.

## Methods

### Data collection and review

The data sets for our study were downloaded from Gene Expression Omnibus (NCBI) database. Our search was administered to include DF, DHF, DSS patient and healthy volunteer samples. Data sets with sample source other than PBMC and samples of patients treated with any form of therapy were excluded. Sample collection was independent of age, gender, race, region and ethnicity. After a thorough review, 4 out of 26 dengue data sets available on GEO database, qualifying our established criteria (GSE43777, GSE51808, GSE17924, and GSE16463)^49^,^50^,^51^,^52^ were retrieved for our study. As these 4 data sets included patient samples with DF and DHF/DSS disease conditions, the data sets were further curated into seven custom data sets, categorizing based on the disease condition of patients. Finally, 4 DF (GSE43777, GSE51808, GSE17924, and GSE16463) and 3 DHF/DSS (GSE43777, GSE51808, and GSE17924) custom data sets were generated for our study. In total, data sets with three different microarray platforms; four Affymetrix, two Agilent, and one Illumina were retrieved, guiding us to implement a global meta-analysis method. Data sets and samples information table can be found in Supplementary Table S1.

### Individual data set analysis

Each data set was individually pre-processed, normalized and analyzed to obtain multiple sets of DEGs. Raw expression data of each data set was downloaded from GEO provided accession links and normalized with a specific normalization method (RMA for Affymetrix, Percentile shift for Agilent and Quantile for Illumina) to remove any systemic variation occurred during microarray experiment. Samples of each data set were classified into two groups, i.e. Diseased (DF or DHF/DSS) and healthy volunteer group. Our analysis of interest included (DF *vs.* HV), (DHF/DSS *vs.* HV). A student’s t-test statistic was performed to check for differential expression of genes (DEG’s) between the two assigned groups. To avoid multiple testing problem, p-values were adjusted using an optimized FDR approach^17^. Genes with adjusted p-values (q-values) less than 0.05 were considered as significant DEGs. In case of repetition of genes (different isoforms) in a list of DEGs, gene isoform with least q-value was taken into account. Later, each gene was annotated with respective Entrez ID., Gene symbol and Gene name. Subsequent to the analyses, seven sets of DEGs were obtained and used for the meta-analysis study. All the above analyses were performed in R using various Bioconductor packages; *GEOquery*, *affy*, *lumi*, *limma*, *annotate*^53^,^54^,^55^.

### Meta-Analysis

The primary purpose of conducting a microarray meta-analysis is to identify a common transcriptional profile of a biological condition in multiple gene expression data sets. From our independent data set analysis, seven sets of DEGs varying in number from hundreds to thousands corresponding to each data set were obtained. In general, meta-analysis method for data sets of same platforms or closely equivalent versions of a platform includes combining q-values of each gene from different data sets prior to the extraction of significant DEGs and assigning the genes with a combined p-value < 0.05 as meta-genes. Unlike the earlier method, here DEGs for each data set were generated separately and those genes which were commonly and consistently expressed across all data sets were considered as meta-genes. For our study, a step by step meta-analysis procedure was as followed:

- DEGs that were commonly expressed in 7 data sets were extracted.
- Common DEGs with inconsistent expression across all data sets were excluded.
- Common DEGs with consistent expression (meta-genes) were re-checked for statistical significance by combining independent q-values of each gene across all data sets using the weighted Z-method^18^.

Our meta-analysis algorithm was implemented in R.

### GO Terms and Pathway Enrichment

Meta-genes obtained from meta-analysis were subjected to GO enrichment analysis and pathway enrichment analysis. Functional roles were annotated based on GO terms (Biological process and molecular function) using Enrichr (http://amp.pharm.mssm.edu/Enrichr/), a web-based online tool^19^. To identify the pathways enriched in the list of meta-genes, ReactomeFIViz^20^, a Cytoscape plug-in based on Reactome pathway database was used.

### *In-vitro* propagation of Dengue viruses

DENV-1 (Hawaii) and DENV-2(TR1751) viral strains used in our study were procured as lyophilized stocks from National Institute of Virology (NIV, Pune, India). DENV-1 and DENV-2 viral stocks were propagated in C636 cells as described elsewhere^56^. The titre of the propagated viruses were calculated by counting viral plaques observed in Focus Forming Assay (FFU) as described elsewhere^57^.

### THP-1 cell culture and dengue virus infection

THP-1 cells were cultured in RPMI-1640 medium containing 10% FBS and 1% penicillin-streptomycin (Invitrogen-Gibco). Briefly, THP-1 cells were washed thoroughly in RPMI 1640 containing 1% FBS and suspended in 1% RPMI 1640 media. Cells were then incubated with DENV-1 or DENV-2 virus for 2 hrs at 37 °C with frequent agitation after 20 min interval to avoid sedimentation of cells. DENV infected cells were washed to remove excess virus, resuspended in RPMI 1640 containing 10% FBS and reseeded in 6- or 12-well plates at 37 °C for different time points (6, 12 and 24 hrs) before they were harvested for RNA extraction.

### Quantitative real-time PCR (RT-qPCR)

Total RNA from DENV infected THP-1 cells were extracted using TRIZOL reagent (Invitrogen). Purified RNA were reverse transcribed into cDNA using VERSO cDNA synthesis kit (Thermo scientific) according to manufacturer’s instructions. 30 ng cDNA from each group was used for RT-qPCR. Quantitative real-time PCR was performed using Mastercycler ep realplex (Eppendorf). The cDNA’s were amplified using Syber-Green Mix (Kappa Biosystems, USA) with gene-specific primers (see Supplementary Table S3) with the following thermal cycler parameters: one cycle of 94 °C for 2 min followed by 40 cycles of 30 s at 94 °C, 30 s annealing at 56 °C and 40 s extension at 68 °C.The relative mRNA expression for each sample was calculated relative to housekeeping gene β-Actin^58^.

### Transient transfection

*In vitro* transient transfection was done using Lipofectamine^®^ 3000 reagent (Invitrogen, USA) according to manufacturer’s instructions. Briefly, Vero cells were transfected with equal concentration of constructs pCAGGS-Patj-myc (a kind gift from Dr Adachi’s lab, Kyoto University, Japan) or pCMV6-CRTAP-Myc-DDK (Origene, Rockville) or only vector controls at 70–80% confluency in low serum OptiMEM media (Gibco, Life technologies), followed by addition of complete media after 6 hrs incubation. Functional analyses were done 24–48 hrs post transfection.

### Detection of Intracellular Virus by Immunofluorescence

Vero cells were seeded on 13 mm coverslips (HiMedia, India) in 24-well tissue culture plates, grown to 50% confluency and transfected with either pCAGGS-Patj-myc or pCMV6-CRTAP-Myc-DDK or vector alone. Further, the transfected cells were infected with DENV-1 or DENV-2 (moi 5) for 18 hrs. Following infection, the cells were fixed in 4% paraformaldehyde, permeabilized with 0.2% TritonX-100 and incubated with anti-dengue monoclonal Ab (GeneTex, USA) for 1 hr at 37 °C. Cells were then washed with 1XPBS and incubated with Alexa Fluor labelled goat anti-mouse IgG (Invitrogen, USA) for 1 hr at 37 °C. Coverslips were mounted in Prolong Gold antifade reagent (Invitrogen, USA), and the cells were scanned with a confocal microscope LSM 510 (Zeiss, Oberkochen, Germany). The images were analyzed using Zeiss LSM5 software and the number of DENV infected cells in a particular field were counted using Image J (NIH) software as described earlier^59^.

### Focus Forming Unit Reduction Assay (FFURA)

Antiviral activity of PATJ and CRTAP genes were evaluated by measuring the reduction in the number of DENV infectious foci after transfection in Vero cells with pCAGGS-Patj-myc or pCMV6-CRTAP-Myc-DDK expressing constructs as described earlier^60^. Briefly, transfected Vero cells were infected with DENV (moi 5) and incubated for 2 hrs for viral adsorption and further overlaid using conditioned growth medium supplemented with 2% FBS and 1.5% carboxymethyl cellulose (CMC). Post incubation, overlay media was carefully removed, washed with 1XPBS, fixed, permeabilized, and stained with an anti-dengue monoclonal antibody (GeneTex, USA) for 1 hr at 37 °C followed by incubation with HRP-linked anti-mouse secondary Ab for 1 hr at 37 °C. Finally, cells were washed with 1XPBS and developed using DAB substrate. Antiviral activities of PATJ and CRTAP proteins were determined by calculating the percentage of foci reduction (%RF) compared against only vector transfected controls using the following formula; RF (%) = (C − T) × 100/C, where, C is the mean of the number of foci from duplicate wells transfected with vector alone and T is the mean of the number of foci from duplicate wells transfected with either pCAGGS-Patj-myc or pCMV6-CRTAP-Myc-DDK as described earlier^60^. Results were represented as the means ± standard error of the mean (SEM) from duplicate assay from three independent experiments.

### Total Collagen Estimation

Collagen estimation in pCMV6-CRTAP-Myc-DDK or only vector transfected Vero cells was carried out using Total Collagen Assay Kit (Bio Vision, USA) according to manufacturer’s instructions. Briefly, equal concentration of lysates were hydrolysed with 100 μl of concentrated HCL at 120 °C for 3 hrs following which they were vortexed and centrifuged at 10000 × g for 3 minutes to remove the precipitate. 10–30 μl of each hydrolysed sample was transferred to a 96 well plate and evaporated to dryness at 70 °C. 100 μl of Chloramine T reagent was added to each sample and incubated at RT for 5 minutes following which 100 μl of the DMAB reagent was added and incubated for 90 minutes at 60 °C. Finally, absorbance was taken at 560 nm in a micro-titre plate. The concentration of collagen was calculated from Collagen I standard curve provided with the kit.

## Additional Information

**How to cite this article**: Afroz, S. *et al*. Transcriptome meta-analysis reveals a dysregulation in extra cellular matrix and cell junction associated gene signatures during Dengue virus infection. *Sci. Rep.* **6**, 33752; doi: 10.1038/srep33752 (2016).

## Supplementary Material

Supplementary Information
