# Supplementary material for: Transcriptome meta-analysis reveals a dysregulation in extra cellular matrix and cell junction associated gene signatures during Dengue virus infection
Source: Sci Rep. 2016 Sep 21;6:33752. doi: 10.1038/srep33752 (PMC5030657; doi:10.1038/srep33752)
Supplement: Supplementary Information [file srep33752-s1.pdf]

Supplementary Information

Transcriptome meta-analysis reveals a dysregulation in extra cellular matrix and cell-junction associated gene signatures during Dengue virus infection.

Sumbul Afroz[1,+], Jeevan Giddaluru[1,+], Mohd. Manzar Abbas[1],and Nooruddin Khan[1,*]

[1]*School of lifesciences, Department of Biotechnology and Bioinformatics, University of Hyderabad, Hyderabad-500046, Telangana, India.*

[*]**Correspondence should be addressed to** : Nooruddin Khan (noor@uohyd.ac.in).

[+]these authors have contributed equally to this work.

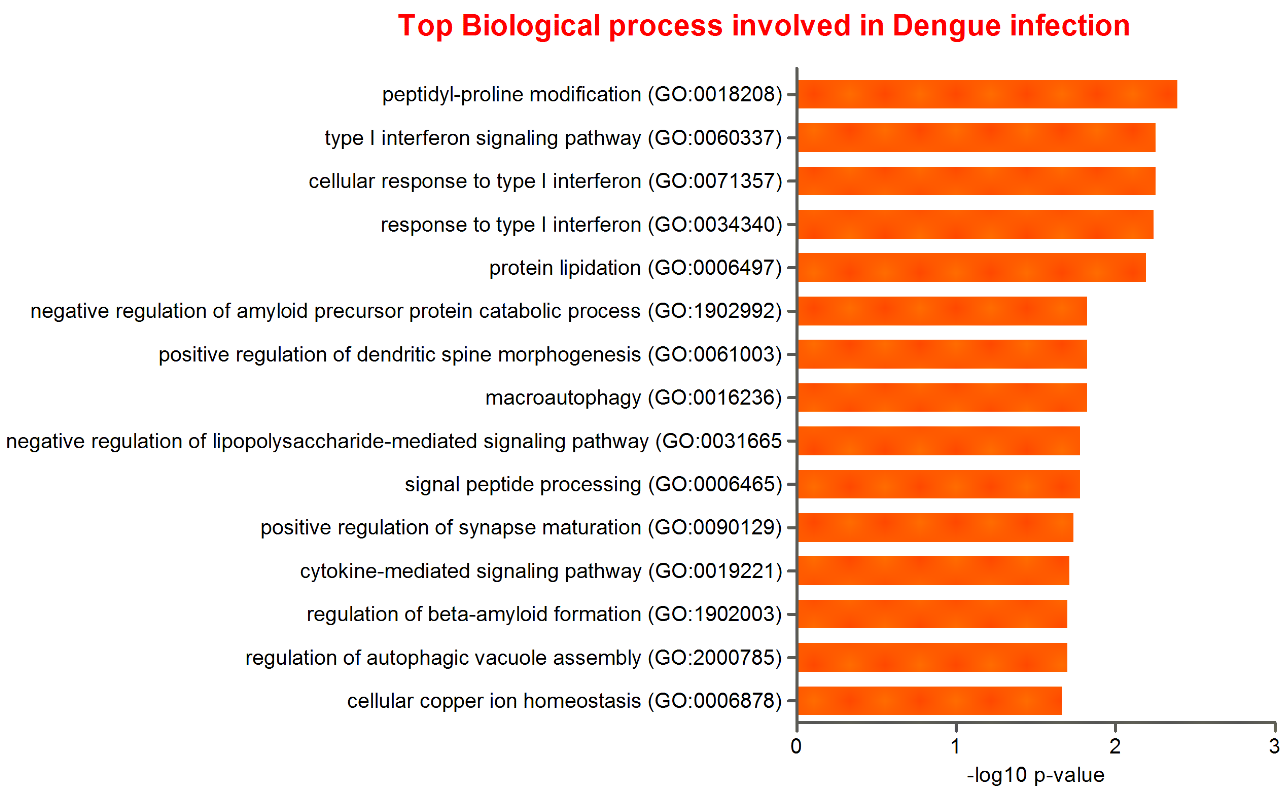

Supplementary Figure S1. Top GO biological process enriched from the set of meta-genes.

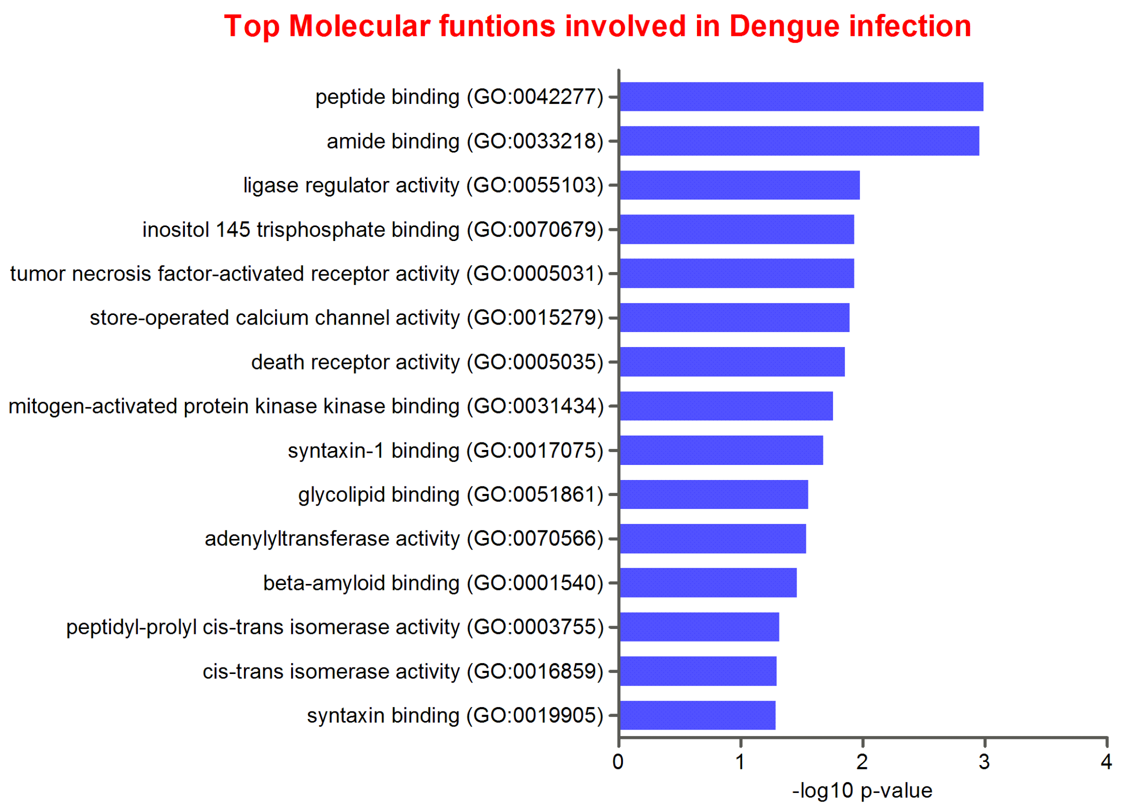

Supplementary Figure S2. Top molecular functions enriched from the set of meta-genes.

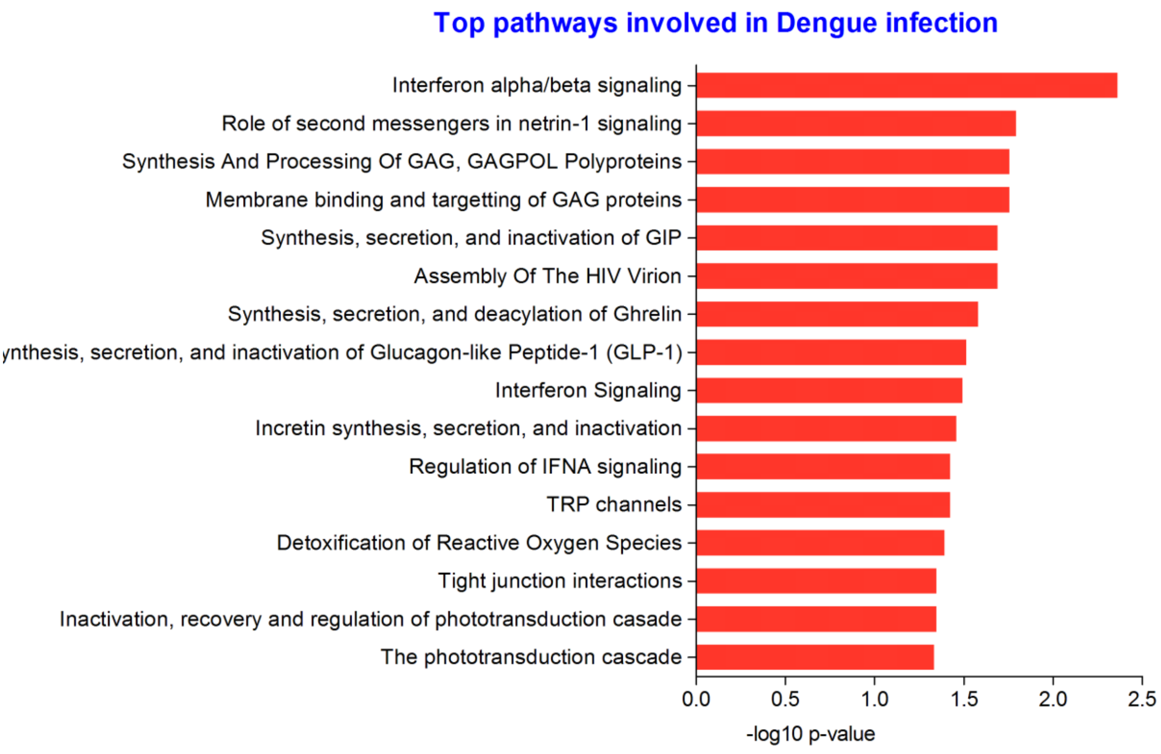

Supplementary Figure S3. Top pathways enriched in Reactome pathway database.

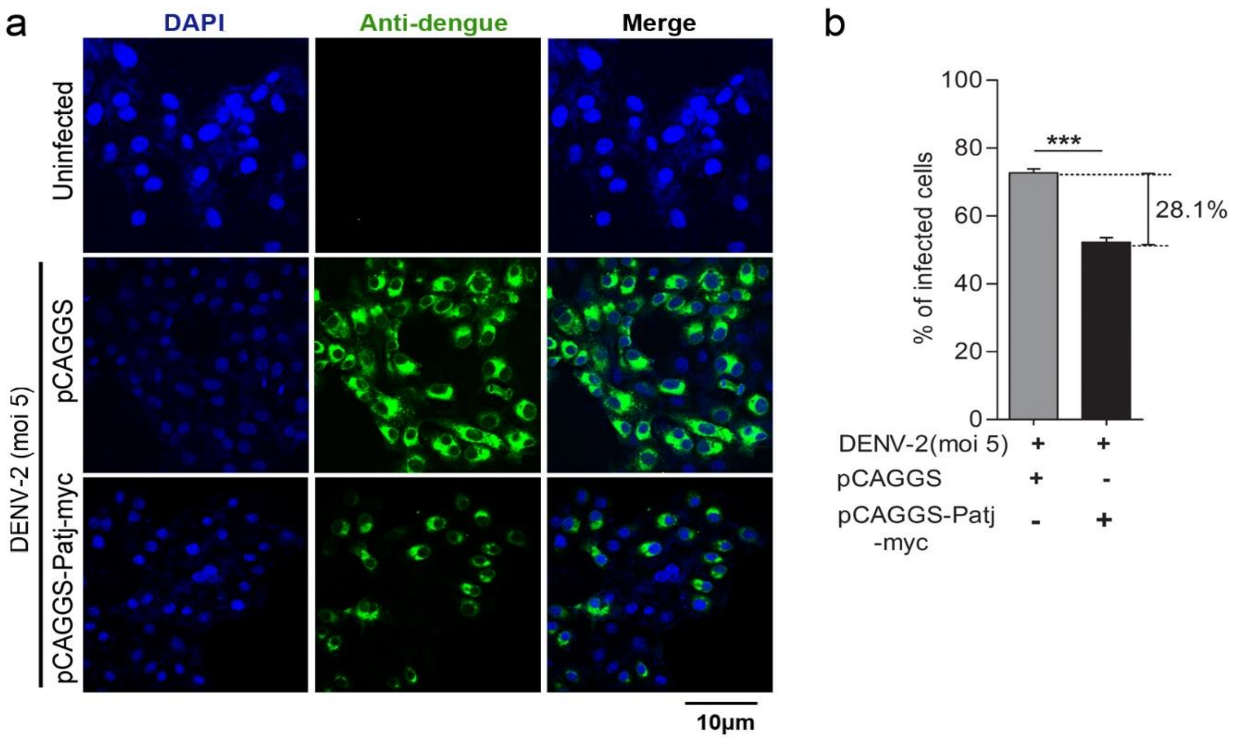

Supplementary Figure S4. PATJ overexpression interferes with DENV-2 infection. a) Laser scanning confocal microscopy to visualize the no. of DENV-1 infected Vero cells transfected with either pCAGGS (vector control) or pCAGGS-Patj-myc. Vero cells were transfected with equal concentrations of both the plasmids for 36 hours followed by infection of DENV-2 (moi 5) for another 18hrs. Following incubation the cells were fixed, permeabilized, and stained with anti-Dengue monoclonal Ab (green). b) Quantification of number of DENV-2 infected cells in vector transfected and pCAGGS-Patj-myc transfected Vero cells. Infected cells were counted in at least ten different fields for each experimental condition using Image J software and average number of infected cells per field were plotted. Statistical analysis was performed using Student's t-test using Graph Pad Prism version 5 (Graph Pad Software Inc., San Diego, CA.). Error bars represent the standard error of the mean (SEM). ***P<0.001, **P<0.01, *P<0.05 were considered statistically significant

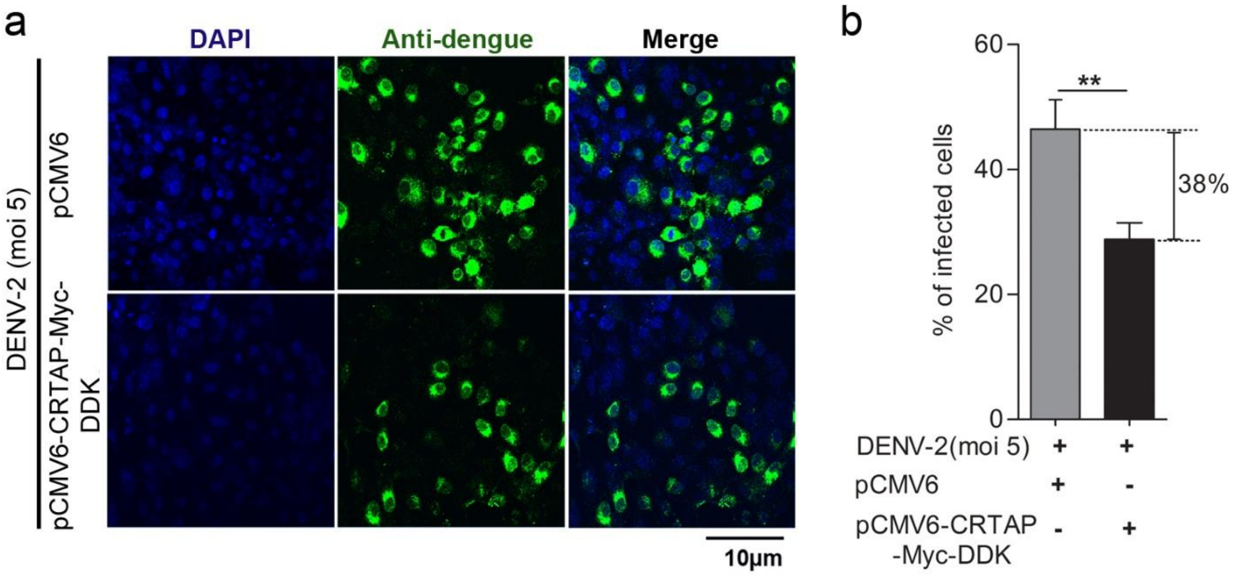

Supplementary Figure S5. Over-expression of CRTAP gene inversely regulates DENV-2 infection. a) Laser scanning confocal microscopy to visualize the number of DENV-2 (moi 5) infected Vero cells transfected with either pCMV6 (vector control) or pCMV6-CRTAP-Myc-DDK. Following incubation the cells were fixed, permeabilized, and stained with anti-Dengue monoclonal Ab (green). b) Quantification of number of DENV-2 infected cells in vector transfected and pCMV6-CRTAP-Myc-DDK transfected Vero cells. Infected cells were counted as mentioned in previous figure. Statistical analysis was performed using Student's t-test using Graph Pad Prism version 5 (Graph Pad Software Inc., San Diego, CA.). Error bars represent the standard error of the mean (SEM). ***P<0.001, **P<0.01, *P<0.05 were considered statistically significant.

Supplementary Table S1. Datasets and samples information.

Custom Dataset ID	GEO Accession ID		Disease Grade	No. of samples		References
	Diseased	Healthy		Diseased	Healthy	
1	GSE43777	GSE18090	DF	25	8	(Sun, García et al. 2013) (Nascimento, Braga-Neto et al. 2009)
2			DHF	24	8	
3	GSE51808	GSE51808	DF	18	9	(Marcin, Nakaya et al. 2014)
4			DHF	10	9	
5	GSE17924	GSE17924	DF	16	52	(Devignot, Sapet et al. 2010)
6			DHF	32	52	
7	GSE16463	GSE16463	DF	7	2	(Tantibhedhyangkul, Prachason et al. 2011)

Supplementary Table S2. **Meta-genes combined p-values and log fold change values.**

Rank	Log Fold Score	Combined p-value (Weighted Z method)	Gene Symbol	Gene Name
1	1.9169512	3.59E-29	CAPRIN1	cell cycle associated protein 1
2	1.750237	2.09E-18	RRBP1	ribosome binding protein 1
3	1.6626014	1.68E-15	RTP4	receptor (chemosensory) transporter protein 4
4	1.4829478	1.68E-20	USP18	ubiquitin specific peptidase 18
5	1.4616151	3.48E-15	OAS2	"2-5-oligoadenylate synthetase 2, 69/71kDa"
6	1.3819559	1.18E-22	SPCS3	signal peptidase complex subunit 3 homolog (S. cerevisiae)
7	1.1588407	1.41E-19	ATOX1	antioxidant 1 copper chaperone
8	1.1382786	4.04E-17	CKAP4	cytoskeleton-associated protein 4
9	1.0105375	1.06E-23	CMC2	C-x(9)-C motif containing 2
10	0.44321185	2.16E-15	TRIB1	tribbles pseudokinase 1
11	-0.38338634	4.90E-15	SPNS3	spinster homolog 3 (Drosophila)
12	-0.39823732	2.05E-19	CTDSP2	"CTD (carboxy-terminal domain, RNA polymerase II, polypeptide A) small phosphatase 2"
13	-0.4850007	8.53E-16	OLFM1	olfactomedin 1
14	-0.52593106	8.81E-21	KIAA1324	KIAA1324
15	-0.54209703	9.03E-18	TGOLN2	trans-golgi network protein 2
16	-0.5644709	5.19E-15	VENTX	VENT homeobox
17	-0.6471077	1.04E-17	NMT2	N-myristoyltransferase 2
18	-0.6885388	2.60E-16	LPHN1	latrophilin 1
19	-0.7059765	1.12E-20	ZNF395	zinc finger protein 395
20	-0.77331513	6.26E-19	TRPC1	"transient receptor potential cation channel, subfamily C, member 1"
21	-0.7881717	2.01E-16	CD1C	CD1c molecule
22	-0.8807664	2.03E-22	RALGPS1	Ral GEF with PH domain and SH3 binding motif 1
23	-0.90612346	1.08E-16	TNFRSF25	"tumor necrosis factor receptor superfamily, member 25"
24	-0.9131341	1.29E-21	CRTAP	cartilage associated protein
25	-0.98072535	5.94E-16	INADL	InaD-like (Drosophila)
26	-1.0732092	1.17E-15	NKTR	natural killer-tumor recognition sequence
27	-1.1038533	1.37E-22	ZCCHC14	"zinc finger, CCHC domain containing 14"
28	-1.1260724	3.84E-16	FCGBP	Fc fragment of IgG binding protein
29	-1.6448973	6.09E-25	STXBP5	syntaxin binding protein 5 (tomosyn)
30	-2.427094	3.38E-18	FNBP1	formin binding protein 1

Supplementary Table S3. Genes specific primers.

SNO.	GENES	PRIMERS
1.	ATOX1	FP: 5’ CGAGAGACAGCTTCAGCACA 3’<br>RP: 5’ GAATCCAGAGAGGCGCTG 3’
2.	OAS2	FP: 5’ TACCATCGGAGTTGCCTCTT 3’<br>RP: 5’ CACTGATCGACGAGATGGTG3’
3.	USP18	FP: 5’AACGTGCCCTTGTTTGTCCAA 3’<br>RP: 5’ GAGTCCTTCACCCGGATCGTA 3’
4.	INADL/PATJ	FP: 5’GAGTGTAGCAGACAGGGATCA 3’<br>RP: 5’TGCTTTTTGTGTGGACTGGTT3’
5.	CRTAP	FP: 5’CAGTTATAGGAGGCTATCCGGT 3’<br>RP: 5’ TCCCTGTGGTACTGGTAATACAC 3’
6.	β-Actin	FP: 5’ CCTTGCACATGCCGGAG 3’<br>RP:5’ GCACAGAGCCTCGCCTT 3’

Supplementary Table S4. Pathway enrichment analysis results.

PATHWAY	P-value	Genes
Interferon alpha/beta signalling	0.004360249	OAS2;USP18
Role of second messengers in netrin-1 signalling	0.016121595	TRPC1
Synthesis And Processing Of GAG, GAGPOL Polyproteins	0.017575531	NMT2
Membrane binding and targeting of GAG proteins	0.017575531	NMT2
Synthesis, secretion, and inactivation of Glucose-dependent Insulinotropic Polypeptide (GIP)	0.020477603	SPCS3
Assembly Of The HIV Virion	0.020477603	NMT2
Synthesis, secretion, and deacylation of Ghrelin	0.026258612	SPCS3
Synthesis, secretion, and inactivation of Glucagon-like Peptide-1 (GLP-1)	0.030574198	SPCS3
Interferon Signalling	0.032133839	OAS2;USP18
Incretin synthesis, secretion, and inactivation	0.034872562	SPCS3
Regulation of IFNA signalling	0.037728601	USP18
TRP channels	0.037728601	TRPC1
Detoxification of Reactive Oxygen Species	0.040577031	ATOX1
Tight junction interactions	0.044835449	INADL
Inactivation, recovery and regulation of the phototransduction cascade	0.044835449	NMT2
The phototransduction cascade	0.046251137	NMT2
Antigen activates B Cell Receptor (BCR) leading to generation of second messengers	0.061699539	TRPC1
Netrin-1 signalling	0.061699539	TRPC1
XBP1(S) activates chaperone genes	0.067261059	CTDSP2
IRE1alpha activates chaperones	0.070030678	CTDSP2
Cytokine signalling in Immune system	0.072777577	OAS2;USP18
Golgi Associated Vesicle Biogenesis	0.078295183	TGOLN2
Cell-cell junction organization	0.086493528	INADL
trans-Golgi Network Vesicle Budding	0.08785352	TGOLN2
Clathrin derived vesicle budding	0.08785352	TGOLN2
Collagen biosynthesis and modifying enzymes	0.091922569	CRTAP
